# Supplementary material for: Complexome profiling of the Chlamydomonas psb28 mutant reveals TEF5 as an early PSII assembly factor
Source: Plant Cell. 2025 Mar 17;37(6):koaf055. doi: 10.1093/plcell/koaf055 (PMC12164590; doi:10.1093/plcell/koaf055)
Supplement: koaf055_Supplementary_Data [file koaf055_supplementary_data.zip › psb28_interactive.zip!/plots/intensity/qualityPlots/peptides/CTH1_Cre12.g510050.t1.2.html]

### Cre12.g510050.t1.2

Trivial name: CTH1  
SeqCoverage [median]: 49.50  
Mass: 47.18 kDa  
  
 

| Sequence | MissedCleavage | Mass [Da] | wtIntensity | muIntensity | Score |
| --- | --- | --- | --- | --- | --- |
| AIMKAPILER | 1 | 1140.67 | 33203.37 | 3103.64 | 120.09 |
| ATNPVVAEIFTLMSR | 0 | 1647.87 | 8612.99 | 0.00 | 122.39 |
| DGIKVASDETLLTPR | 1 | 1613.86 | 9388.69 | 1098.92 | 62.38 |
| ESGSYDLDANK | 0 | 1197.52 | 16679.18 | 1636.26 | 194.06 |
| ESGSYDLDANKTALVY | 1 | 1744.82 | 9943.16 | 0.00 | 147.63 |
| FYTTDFDEMER | 0 | 1452.59 | 25151.21 | 4998.72 | 163.88 |
| HFVRNETFKEAAEK | 2 | 1704.86 | 776.20 | 0.00 | 65.89 |
| HGDFFTAVLK | 0 | 1133.59 | 717.21 | 0.00 | 82.45 |
| IFPAVPDVENPEFFR | 0 | 1775.89 | 6292.00 | 0.00 | 113.22 |
| IFPAVPDVENPEFFRR | 1 | 1931.99 | 7256.78 | 0.00 | 57.80 |
| LDYNQR | 0 | 807.39 | 1981.62 | 0.00 | 126.04 |
| LFSLELNK | 0 | 962.54 | 738.97 | 0.00 | 63.56 |
| MVAEVFQVFIMTPK | 0 | 1638.85 | 3551.93 | 0.00 | 149.49 |
| NETFKEAAEK | 1 | 1165.56 | 10270.03 | 0.00 | 83.95 |
| VASDETLLTPR | 0 | 1200.63 | 44827.63 | 14068.81 | 163.64 |
| YISIYR | 0 | 813.44 | 7473.24 | 0.00 | 119.23 |
| YNAQLVNIGSMNLPSPIK | 0 | 1958.03 | 16674.73 | 0.00 | 195.38 |
|  |  |  | 203538.9 | 24906.4 |  |
